# Using advanced vibrational molecular spectroscopy to detect moist heating induced protein structure changes in cool-climate adapted barley grain

**DOI:** 10.1371/journal.pone.0234126

**Published:** 2020-06-05

**Authors:** Xin Feng, Baoli Sun, Huihua Zhang, Peiqiang Yu

**Affiliations:** 1 Department of Animal and Poultry Science, College of Agriculture and Bioresources, University of Saskatchewan, Saskatoon, SK, Canada; 2 School of Life Science and Engineering, Foshan University, Foshan, China; 3 College of Animal Science, South China Agricultural University, Guangzhou, China; Luleå University of Technology, SWEDEN

## Abstract

Different techniques have been applied in feed processing to improve ruminal degradation and nutrient utilization in ruminant. There are limited studies investigating how moist heating process impacts barley protein utilization and internal molecular structures. The objectives of this study were to investigate: 1) how moist heating affects barley protein chemical profiles and Cornell Net Carbohydrate and Protein System (CNCPS) subfractions, in situ rumen degradation parameters, and predicted intestinal protein supply and feed milk value; 2) how moist heating affects protein molecular spectral features; and 3) the relationship between protein molecular structure spectral features and protein chemical profiles and metabolic characteristics. The barley variety CDC cowboy samples collected from the research farm during two consecutive years were used. Half of each sample was kept as raw and the other half underwent moist heating. The advanced molecular spectroscopy (attenuated total reflectance-fourier transform infrared, ATR-FTIR) was used to detect the barley protein molecular structure spectral features. It was found that moist heating decreased the fractions of soluble protein and increased the moderately degradable protein and ingestible protein fractions. This further resulted in the changes of in situ rumen degradation parameters and intestinal protein digestion characteristics. The protein molecular structure spectral features detected by using ATR-FTIR spectroscopy can be used as potential predictors for protein related chemical and metabolic parameters.

## Introduction

Barley grain (*Hordeum vulgare L*.) is the primary energy and protein source for beef and dairy cattle diets in western Canada. Animal nutritionists have been working closely with plant breeders to identify barley variety with the most desirable nutritional characteristics for ruminants. Barley has higher neutral detergent fiber (NDF) and acid detergent fiber (ADF) content relative to corn and wheat [1]. Compared to corn, the higher protein content of barley is offset by somewhat lower energy content due to its higher fiber content. In addition, the lysine, methionine, cysteine and tryptophan in barley are also higher compared to corn [2]. The higher protein content of barley can save costs of supplemental protein but the fact that most of protein is degradable in the rumen is a disadvantage. Barley has a higher rate of ruminal starch degradation which may influence the ammonia utilization rate by altering the energy supply for microbial growth [3]. Two lactating cow studies did not observe a difference of ruminal synchronization of energy and protein when cows were fed corn or barley as starch sources [4, 5].

For ruminants, the rumen is the first site where the nutrients are being digested by the ruminal microbes. The extent and amount of nutrients digested in the rumen are affected by grain type, processing method, nutrient composition, and passage rate [2]. Barley ferments rapidly in the rumen compared to some other grains. The rapid fermentation poses a greater risk of acidosis and also causes lower nitrogen utilization. Physical or chemical processing affects the animals’ response to the grains fed. Barley processing requires careful attention to maximize digestion efficiency and maintain stable rumen function. The commonly used processing methods for barley include rolling, tempering, steam-flaking, roasting, and pelleting [1]. Compared to dry rolling, steam rolling could increase the moisture content of barley, resulting in less fine particles which further reduces the risks of rumen acidosis [1]. Similar to flame roasting, high pressure heating could decrease the degradation rate of barley in the rumen without affecting the total tract digestibility [6].

Heat treatment has been used as an efficient method for reducing some anti-nutritional factors such as tannin, protease inhibitors, alkaloids among others [7]. To some extent, heat processing is able to change the physical and nutritional characteristics, thus resulting in slower degradation of starch and protein in the rumen and maximizing the utilization of these nutrients. It was reported by Sadeghi et al. [8] that heat treatment can break the covalent and non-covalent bonds thus rendering the denatured protein structure more resistant to the degradation of rumen microbes. It is generally accepted that animals fed processed barley performed better than those fed whole barley. In beef cattle, dry-rolled barley had a dry matter digestibility of 85.2% whereas the whole barley only had 52.5%. Compared to the control group cows, the rolled barley group cows had higher milk yield but the alkali-treated group was not different [9]. Orskov et al. [10] noted that the alkali-treated barley improved utilization to a greater extent than coarse rolled barley.

Content and composition of feed and the physical structure of protein molecule both affect the protein utilization in animals. Yu et al. [11] reported that ruminal degradation characteristics and post-ruminal protein availability were affected by the intrinsic molecular structure of the feed protein. Understanding protein digestibility and utilization through protein intrinsic molecular structures could be a potential method to replace the traditional chemical analysis and animal trials. In the traditional chemical analysis, harsh chemicals are often used which can destroy the internal structure of the samples, whereas the feed protein evaluation by animal trials is time consuming and not so cost effective. Attenuated total reflectance Fourier transform infrared spectroscopy (ATR-FTIR) is a rapid, direct, and non-destructive technique for analyzing the molecular structures of nutrients [11].

The objectives of this study were to investigate: 1) how moist heating affects barley protein chemical profiles and Cornell Net Carbohydrate and Protein System (CNCPS) subfractions, in situ rumen degradation kinetics, and predicted protein supply; 2) how moist heating affects protein molecular spectral features; and 3) the relationship between protein molecular structure spectral features and protein chemical profiles as well as metabolic characteristics.

## Materials and methods

The animal study protocol was approved by the Animal Care Committee of the University of Saskatchewan (Protocol number: 19910012). Animals used for studies were cared for in accordance with the guidelines of the Canadian Council on Animal Care. [12]

### Samples and treatment

CDC cowboy (*Hordeum vulgare*) is a barley variety made in 1993 by the breeder Brain Rossnagel of the Crop development Center (CDC; University of Saskatchewan, Saskatoon, Canada). This variety was selected based on the criteria such as high forage yield and quality, physiological maturity, plant height, and disease resistance. Two samples were collected from Kernen Crop Research Farm (University of Saskatchewan, Canada) during two consecutive years. Then each sample was divided into two portions. One portion of each sample was kept as raw (Control) and the other portion underwent pressure heating (Moist heating with saturated steam) in an autoclave (Amsco Eagle Sg-3031, Steris Corp., Mentor, OH, USA) at 120 Cº for 60 min (Moisture heating). The samples were cooled to room temperature (20–22 Cº) before being ground through a roller mill with a 1.499 mm gap (Sven Grain Mill, Apollo Machine and Products, Saskatoon, Canada) for in situ rumen fermentation. Portion of each sample from the roller mill was further ground through in a Retsch mill (Retsch ZM-200, Brinkmann Instruments Ltd., Mississauga, ON, Canada) for chemical analysis and intestinal digestion (1 mm screen), and molecular spectral analysis (0.12 mm screen).

### In situ rumen fermentation

For in situ rumen fermentation, four cannulated lactating cows were used. The cows were kept in individual stalls at the Rayner Dairy Research and Teaching Facility of the University of Saskatchewan (Saskatoon, Canada). During the trial, the cows had free access to total mixed ration (TMR) and drinking water. The TMR was formulated based on NRC (2001) [13] including 45.8% barley silage, 12.1% hay, and 31.3% concentrate.

The standard nylon bag technique was used for in situ rumen fermentation. The detailed method including the nylon bag parameters, incubation intervals, the “gradual addition/all out” schedule, incubation replicates/runs, and post-incubation handling were all the same as reported previously [14]. The first-order kinetics model by Orskov et al [15] and Tamminga et al. [16] was used to estimate ruminal crude protein degradation kinetics:

$$R\left(t\right)=U+D\times exp^{\left[‐kd(t‐T0)\right]}$$

where R(t) is the incubation residue (%) after t h, U is the undegradable fraction (%), D is the potentially degradable fraction (%, D = 100-S-U), K_d_ is degradation rate (%/h), and T_0_ is lag time (h).

### Chemical analysis

The feed samples were analyzed for dry matter (DM; 930.15), crude protein (CP; 984.13), ash (942.05), ether extract (EE; 920.39) according to AOAC methods [17]. The total carbohydrate (CHO) content was calculated as CHO = 100- EE-CP-ash according to the NRC (2001). The rumen fermentation residues were ground through the Retsch mill (1 mm screen) before chemical analysis. The non-protein nitrogen (NPN), neutral detergent insoluble protein (NDICP), and acid detergent insoluble protein (ADICP) were determined following the method reported previously [18]. Soluble protein (SCP) was analyzed according to Roe et al. [19] with minor modifications [20]. The rumen degraded protein (RDP) and rumen undegraded protein were estimated with an assumed passage rate Kp of 6% /h [16] and the following equations:

RDP(%)=SCP+D×[Kd/(Kd+Kp)]

RUP(%)=U+D×[Kp/(Kd+Kp)]

### Protein subfractions and intestinal digestion of RUP

The CP subfractions were partitioned using the Cornell Carbohydrate and Protein System (CNCPS 6.5) [21]. The subfractions include: PA1 where PA1 = ammonia, PA2 (soluble true protein) where PA2 = SCP-PA1, PB1 (insoluble true protein) where PB1 = CP–(PA1 +PA2 + PB2 + PC), PB2 (fiber-bound protein) where PB2 = (NDICP-ADICP), and PC (ingestible true protein) where PC = ADICP. The degradation rates (Kd) for PA1, PA2, PB1, and PB2 are 200%/h, 10–40%/h, 3–20%/h, and 1–18%h [22].

Intestinal digestibility of RUP (IDP, %RUP) was determined using the modified three-step in vitro procedure [23]. The intestinal digestibility of RUP was calculated as percent of RUP and total CP (IDP, %CP), respectively. The total digestible crude protein (TDP, %CP) was the summation of RDP (%CP) and IDP (% CP).

### Metabolizable protein and feed milk value estimation

The metabolizable protein (MP) supply to dairy cows from barley grain was estimated. Briefly, the total MP supply to dairy cattle is the summation of truly absorbable rumen synthesized microbial protein (AMCP), truly absorbable RUP (ARUP), and truly absorbable endogenous protein supply to the small intestine (AECP). The degraded protein balance (DPB) was estimated as the difference between the potential MCP synthesized based on RDP and TDN. All the model equations as well as the coefficients used for calculation are in accordance with the NRC (2001). The feed milk value (FMV) is the efficiency of true feed protein for milk production and it was calculated as FMV = MP ×0.67×0.033 where 0.67 is the conversion efficiency and 0.033 is the milk protein content (g/g) [20].

### Protein molecular spectra collection and analysis

The protein molecular spectral profiles were collected using a JASCO FT/IR-ATR-4200 spectroscope (JASCO Corp., Tokyo, Japan) at the molecular spectroscopy lab (University of Saskatchewan, Saskatoon, Canada). The spectroscope was equipped with a MIRacle ATR accessory module, a ZnSe crystal and pressure clamp (Pike Technologies, Madison, WI). Spectra were collected from the mid-infrared range from *ca*. 4000–700 cm^-1^ with 256 scans per spectrum at 4 cm^-1^ resolution. The protein related molecular spectral peak bands were identified according to the published literature [24] including: amide I (region *ca*. 1723~1588 cm^-1^; peak *ca*. 1644 cm^-1^), amide II (region *ca*. 1588~1482 cm^-1^; peak *ca*. 1539 cm^-1^), α-helix (*ca*. 1650 cm^-1^) and β-sheet (*ca*. 1637 cm^-1^). Different peak area and height intensity ratios were also calculated in this study. The OMNIC 7.3 (Thermo Electron Corp., Madison, WI, US) was used for quantification of absorption peaks areas and heights of spectral bands related to protein primary and secondary structures. The principle component analysis (PCA) was performed on the overall spectral data related to protein structures (*ca*. 1723~1482) to visualize the overall difference in protein molecular structures between raw barley and moist-heated barley. The detailed information regarding PCA for ATR-FTIR spectral data are described in Yu (2007) [24].

### Statistical analysis

The protein chemical composition, protein subfractions, intestinal digestibility of RUP, estimated protein supply, and molecular structure spectral parameters were analyzed using the PROC MIXED procedure of SAS (SAS Institute, Inc., Cary, NC, USA) with the heating treatment as fixed effect. Because there were two in situ runs for rumen fermentation, the rumen degradation parameters data was analyzed using the PROC MIXED procedure of SAS with the heating treatment as fixed effect and the in situ run as random effect. The difference was declared significant at *P* < 0.05 and trends at *P* < 0.1.

For the regression analysis only variables contributing significantly (*P* < 0.05) to the dependent variable were retained in the model. Model assumptions were checked through the residual analysis and significance was declared at *P* < 0.05.

## Results and discussion

### Protein chemical profiles and subfractions

The effects of moist heating on protein chemical profiles and CNCPS subfractions of barley are presented in Table 1. The CP and NPN were not affected by the heat treatment whereas SCP tended to be lower in the heat treated barley compared to the raw sample (1.43 vs. 3.39; *P* = 0.056). Compared to the control group, the heat treatment increased the content of ADICP (*P* = 0.012) with a trend to increase NDICP. Nikkhah et al. [25] reported heating effectively reduced the soluble fraction of protein and increased the more slowly degradable fraction, which is similar to the current study. Regarding protein CNCPS subfractions, the PA2, which is the soluble true protein, was lower in the heat treated group compared to the control group as expected. The insoluble protein and fiber bound protein, which is PB1 and PB2, both tended to increase after moist heating (*P* = 0.062; *P* = 0.08). Moist heating significantly increased the PC content, which is known as the ingestible protein (*P* = 0.003). Among the many factors affecting ruminal degradation and intestinal digestion of protein, the chemical composition is the most important factor as it is directly related to the digestion and utilization in animals[1]. In the current study, moist heating increased the content of slowly-degradable protein. The chemical reactions, such as the Maillard reaction and denaturation during heating process could be the reason [26]. This also, to some extent, reduced the nitrogen loss in the rumen, thus maximize protein utilization in the animals. Overall, protein chemical composition and CNCPS subfractions showed a similar trend under moist heating. Moist heating treatment reduced the rapidly degradable protein resulting in a higher supply of RUP entering the post-ruminal tract.

**Table 1:** Effects of moist heating on protein chemical profiles and CNCPS subfractions of barley grain.

Item	Treatment		SEM	*P* value
	Control	Moist Heating		
Protein profile (% DM)				
CP	12.96	12.97	0.892	0.987
NPN	0.74	1.00	0.175	0.157
SCP	3.39	1.43	0.129	0.056
ADICP	0.02	0.37	0.027	0.012
NDICP	1.19	2.14	0.175	0.061
Protein subfractions (CP %)				
PA2	26.18	10.33	0.506	0.005
PB1	64.26	71.17	1.044	0.062
PB2	9.45	15.66	0.717	0.080
PC	0.11	2.83	0.116	0.003

SEM: standard error of means. DM: dry matter; CP: crude protein; NPN: non-protein nitrogen; SCP: soluble crude protein; ADICP: acid detergent insoluble crude protein; NDICP: neutral detergent insoluble crude protein; PA2: rapidly degradable true protein (soluble true protein); PB1: moderately degradable true protein; PB2: slowly degradable true protein (bound in NDF); PC: ingestible protein

### In situ rumen degradation kinetics, intestinal digestion, and predicted protein supply

A summary of moist heating effects on in situ rumen degradation parameters and predicted protein supply in the intestine are presented in Table 2 and Table 3. Compared to raw barley, moist heating reduced the degradation rate of the degradable fraction of protein in the rumen (*P* = 0.002). Consequently, the RUP was higher in the moist heating group than that in the control group (69.78 vs 41.52; *P* < 0.001). The undegradable fraction (U) was higher in the control group than the heat treatment group. At the current heating condition, the moist heating decreased degradation rate and reduced U fraction. Although the U fraction was higher, the control group still had lower RUP due to the high degradation rate (9.97 vs. 2.61; *P* = 0.002). As a percent of RUP, intestinal digestible protein was not different between the two groups. However, when intestinal digestible protein was expressed as percent of CP, the heat treatment group was significantly higher than control group (*P* < 0.001). The moist heated barley had a higher content of RUP resulting in less microbial protein synthesis and lower truly absorbed microbial protein as well as higher truly absorbed RUP in the small intestine (*P* < 0.01). In addition, moist heating increased metabolizable protein supply and feed milk value (*P* < 0.01; Table 3).

**Table 2:** Effects of moist heating on in situ rumen protein degradation parameters of barley grain.

Item	Treatment		SEM	*P* value
	Control	Moist Heating		
Kd (%/h)	9.97	2.61	1.244	0.002
S (%)	1.30	0.00	0.785	0.200
D (%)	93.32	100.0	2.049	0.068
U (%)	5.38	0.00	1.519	0.046
RUP (%CP)	41.52	69.78	2.709	< .0001
RDP (%CP)	58.47	30.22	2.709	< .0001

SEM = standard error of mean. Kd: the rate of degradation of D fraction (%/h); S: soluble fraction in the in situ incubation; D: degradable fractions; U: undegradable fractions; RUP: rumen undegradable protein; RDP: rumen degradable protein

**Table 3:** Effects of moist heating on intestinal protein digestion and predicted intestinal protein supply of barley grain using NRC (2001).

Item	Treatment		SEM	*P* value
	Control	Moist Heating		
Intestinal protein digestion				
IDP (% RUP)	69.15	68.57	1.821	0.829
IDP (% CP)	28.63	47.81	1.461	< .0001
Predicted intestinal protein supply (g/kg DM)				
AMCP	41.43	21.34	4.089	0.000
ARUP	15.36	43.06	0.825	< .0001
AECP	4.25	4.34	0.028	< .0001
MP	61.04	68.74	3.854	0.001
DPB	-44.29	-78.88	6.900	0.000
FMV (kg milk/kg feed)	1.24	1.40	0.079	0.000

SEM: standard error of mean. IDP: intestinal digestible protein; AMCP: truly absorbed microbial protein in the small intestine; ARUP: truly absorbed rumen undegradable protein in the small intestine; AECP: truly absorbed rumen endogenous protein in the small intestine; MP: metabolizable protein. DPB: degraded protein balance; FMV: feed milk value

Grain processing can affect the digestion rate and extent, as well as the place where nutrients are digested in the animal [27]. Nikkhah et al. [25] reported that effective ruminal protein degradation of microwave irradiated barley grain was lower compared to raw barley. The author also found that the longer the irradiation time, the lower the effective degradation in the rumen. During the heating process, the unfolding and denaturation of protein lowered the three-dimensional structure stability while breaking the bonds and transformed the proteins to a structure more resistant to enzyme [28]. Prestlokken et al. [29] pointed out that heat treatment degraded the hydrophobic amino acid to a lesser extent than hydrophilic amino acids, whereas more hydrophobic groups exposure could reduce the protein solubility [26]. This could be one of the reasons that the moist heating group had a lower degradation rate compared to the control group. Barley has one of the fastest degradation rates among the commonly used grains, preceded only by dry-rolled wheat [1]. Replacing the coarse rolled barley with highly processed barley increased the microbial protein flow to the duodenum because of the lower degradation rate of coarsely barley [30]. Similar to the current study, the heated barley had lower AMCP due to the lower ruminal degradation rate after heat processing.

### Protein molecular structure spectral features

**Fig 1:**
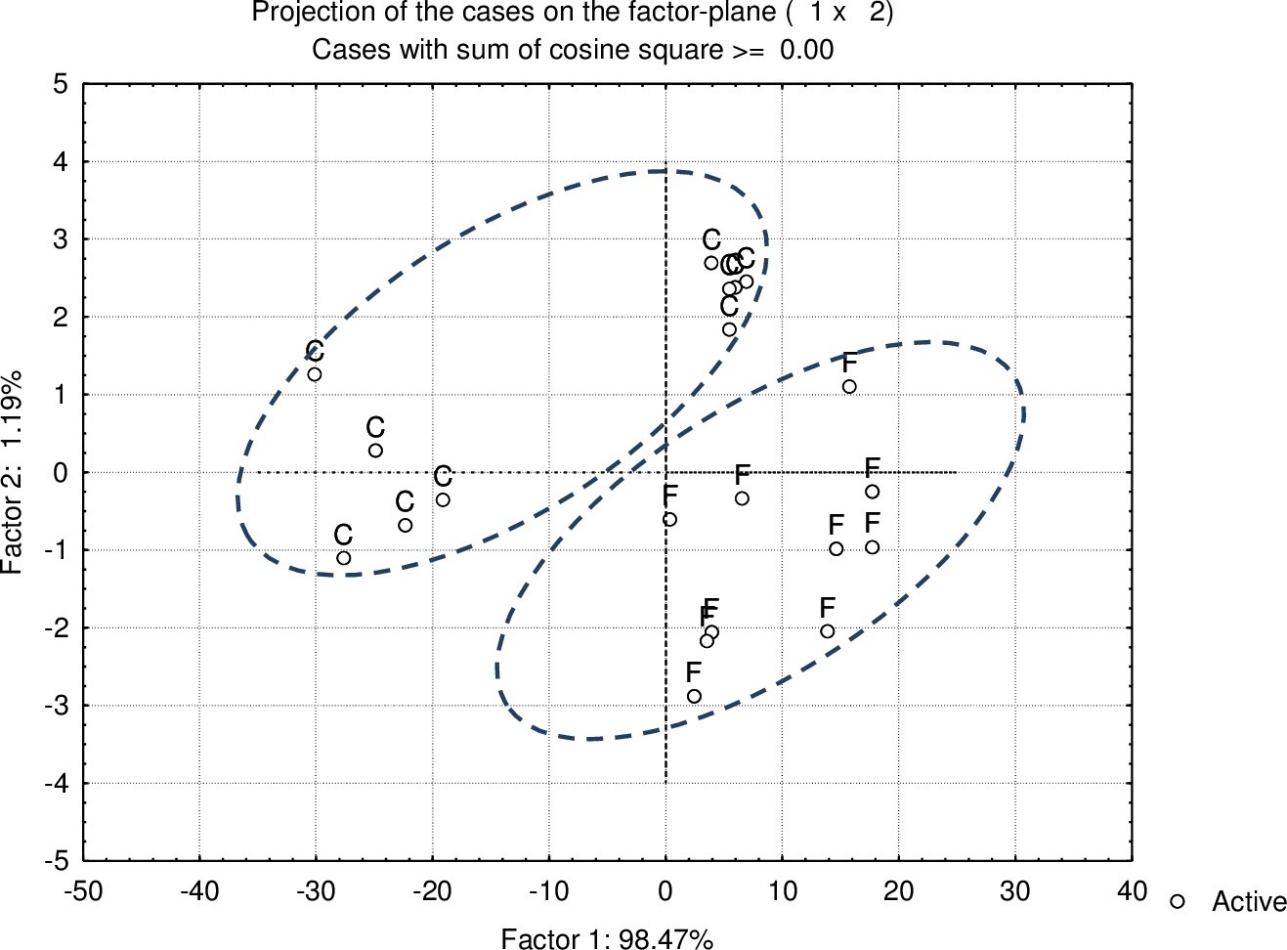
Multivariate molecular spectral analyses of barley (C) and Heated barley (F) at ATR-FTIR protein fingerprint region: Ca. 1723–1482 cm^-1^ using PCA (principal component analysis): Scatter plots of the 1^st^ principal components (PC1) vs. the 2^nd^ principal components (PC2).

The effects of moist heating on the protein molecular structure spectral characteristics are presented in Table 4. Reported spectral features included the amide I area and height, amide II area and height, area ratio of amide I to amide II, and protein secondary structure α-helix and β-sheet. The moist heating did not affect the intensity of amide I area/height and amide II area/height. However, it tended to increase the area ratio of amide I to amide II. The protein secondary structure, α-helix and β-sheet, were identified using the second derivative spectrum and neither of the two was affected by moist heating. However, their height ratio was increased by moist heating. The multivariate molecular spectral analysis at the protein fingerprint region (*ca*. 1723–1482 cm^-1^) for the two treatment groups were present in Fig 1 and the principal component analysis can clearly identify the two groups (Fig 1). The first principal component and second principal component explained 98.47% and 1.19% variation, respectively. The molecular structure of the feed protein affects ruminal protein degradation and further intestinal digestion of RUP by exerting impact on protein solubility and accessibility to microbes and proteolytic enzymes [14]. The ratio of amide I and amide II reveals the differences of the protein molecular structure and biological tissues. Additionally the secondary structure of protein affects the nutritive value and quality of protein. Thus the difference of α-helix to β-sheet ratio indicates different nutritive value and protein availability [24]. The raw barley and heat treated barley did not differ in amide I height/area, amide II height/areas, and the ratios. This indicates the similarity in protein molecular structure. However, the multivariate analysis (PCA) clearly showed the structural differences. whereas the ruminal degradation parameters were also different between two treatment groups. These findings imply that the presented spectral features may not be the only indicators of protein molecular structure.

**Table 4:** Effects of moist heating on protein molecular structure spectral features of barley grain.

Item	Peak region and peak (cm^-1^)	Treatment		SEM	*P* value
		Control	Moist Heating		
Protein primary structure					
Amide I area	1723~1588	16.00	12.54	1.420	0.165
Amide II area	1588~1482	6.00	3.76	1.185	0.252
Amide I peak height	~1644	0.22	0.17	0.019	0.165
Amide II peak height	~1539	0.10	0.07	0.015	0.210
Area ratio of Amide I:II		2.78	3.39	0.376	0.094
Protein secondary structure					
α-helix peak height	~1650	0.22	0.17	0.018	0.157
β-sheet peak height	~1637	0.22	0.15	0.018	0.139
Height ratio of α-helix: β-sheet		1.02	1.13	0.003	0.002

SEM: standard error of mean.

### Regression prediction equations using protein spectral features

The protein molecular structure characteristics were used to predict the protein related characteristics. The predicted protein characteristics include protein chemical profiles, subfractions, intestinal protein digestion and supply. Only variable with *P* < 0.05 was retained in the model and the regression prediction equations are presented in Table 5. For most of the dependent variables, only peak height ratio of α-helix to β-sheet was retained in the model. The possible reason may be that moist heating treatment did not affect most of the protein molecular spectral features as shown in Table 4 [11]. For PB1, moderately degradable true protein, peak height of β-sheet was left in the model. For milk feed value, both area ratio of amide I to amide II and peak height of α-helix were retained in the model. All the prediction equations listed in the table had a R2 greater than 0.94, indicating a high power of prediction ability. As a fully established, non-destructive analytical technique, ATR-FTIR molecular spectroscopy can analyze a large number of samples in a short time [11]. With the relatively good estimation power, the molecular spectral characteristics are potential predictors for predicting nutrient value. It is warranted that the prediction equations listed are based on a small number of samples. A large number of samples from different sources need to be analyzed and modeled to illustrate the relationship between protein molecular structure features and protein related parameters.

**Table 5:** Multiple regression equations for predicting protein profiles, protein subfractions, rumen protein degradation kinetics, intestinal protein digestion and true protein supply using protein molecular structure spectral features.

Predicted variables	Prediction Equations			Model R^2^ value	RSD	*P* value
Protein profiles (% DM)						
CP	NA					
SCP	20.89–17.14×H_AB			0.98	0.22	0.012
ADICP	–3.03 +2.99×H_AB			0.96	0.05	0.021
NDICP	NA					
Protein subfractions (% CP)						
PA2	167.29–138.18×H_AB			0.99	1.21	0.006
PB1	84.41–89.87×H_B			0.96	1.01	0.020
PB2	– 45.99+54.28×H_AB			0.94	1.08	0.029
PC	– 24.06+23.67×H_AB			0.98	0.24	0.008
In situ rumen degradation						
S (%)	NA					
D (%)	NA					
U (%)	NA					
RUP (%CP)	–210.84 +247.10×H_AB					
RDP (%CP)	310.84–247.10×H_AB					
Intestinal protein digestion						
IDP (% CP)	–143.00+168.04×H_AB			0.98	1.77	0.008
Predicted MP and DPB(g/kg DM)						
MP	NA					
DPB	NA					
FMV (kg milk/kg feed)	1.03+0.16×A_AI_II–1.12×H_A			1.00	0.00	0.000

Protein molecular structure spectral parameters: A_AI_II: area ratio of amide I to amide II; H_A: peak height of α helix; H_B: peak height of β sheet; H_AB: peak height ratio of α helix to β sheet. RSD = Residual standard deviation. CP: crude protein; NPN: non-protein nitrogen; SCP: soluble crude protein; ADICP: acid detergent insoluble crude protein; NDICP: neutral detergent insoluble crude protein; PA2: rapidly degradable true protein; PB1: moderately degradable true protein. PB2: slowly degradable true protein; PC: ingestible protein; IDP: intestinal digestible protein; TDP: total intestinal digestible protein; MP: metabolizable protein; DPB: degraded protein balance; FMV: feed milk value; NA: no variable left in the model with the *P* < 0.05 criteria.

## Conclusions

Moist heating decreased the fractions of soluble protein and increased the moderately degradable protein as well as ingestible protein fractions. It further resulted in the changes of in situ rumen degradation parameters and intestinal protein digestion characteristics. The protein molecular structure spectral features detected by using ATR-FTIR spectroscopy can be used as potential predictors for protein related chemical and metabolic parameters.
